# Effect of *Lacticaseibacillus paracasei* N1115 on Immunomodulatory and Gut Microbial Composition in Young Children: A Randomized, Placebo-Controlled Study

**DOI:** 10.3390/nu15081970

**Published:** 2023-04-19

**Authors:** Pin Li, Zhongxia Ren, Junxiu Zhou, Ai Zhao, Shijie Wang, Yiping Xun, Hua Jiang, Peiyu Wang, Qingbin Yuan, Yumei Zhang

**Affiliations:** 1 Department of Nutrition and Food Hygiene, School of Public Health, Peking University, Beijing 100191, China; 2111110222@bjmu.edu.cn (P.L.);; 2 Qilu Hospital, Cheloo College of Medicine, Shandong University, Jinan 250012, China; 3 Vanke School of Public Health, Tsinghua University, Beijing 100084, China; 4 Shijiazhuang Junlebao Dairy Co., Ltd., Shijiazhuang 050221, China; 5 The Joint Laboratory of Human Milk Research & Life Science by the Health Science Center of Peking University and the Junlebao Dairy Group, Beijing 100191, China; 6 School of Nursing, Peking University, Beijing 100091, China; jianghua@bjmu.edu.cn; 7 Department of Social Medicine and Health Education, School of Public Health, Peking University, Beijing 100191, China

**Keywords:** probiotic, young children, cesarean section, *Lacticaseibacillus*

## Abstract

*Lactobacillus paracasei* N1115 (Lp N1115) was isolated from fermented milk products. The administration of Lp N1115 is safe and well tolerated in Chinese children, but its effectiveness among young Chinese children is still unclear. To investigate the efficacy of Lp N1115 as a probiotic to enhance gut development in Chinese infants and toddlers born by cesarean section, 109 healthy and cesarean-delivered infants aged 6–24 months were recruited for a 12-week randomized, placebo-controlled trial, with 101 finally completing the study. Saliva and stool samples were collected and detected at weeks 0, 4, 8, and 12 of the intervention. Statistical analyses were performed by using a per-protocol (PP) approach. After 12 weeks of intervention, the fecal pH in the control group increased (*p* = 0.003), while the fecal pH in the experimental group did not change. Salivary cortisol decreased from baseline in the experimental group (*p* = 0.023), while the control group showed little change. In addition, Lp N1115 increased the fecal sIgA content of infants aged 6–12 months (*p* = 0.044) but had no obvious effect on fecal calprotectin and saliva sIgA. At week 4, the increase in *Lactobacillus* relative to baseline was higher in the experimental group than in the control group (*p* = 0.019). Further analysis showed a trend toward a higher detection rate of *Lactobacillus* in the experimental group than in the control group (*p* = 0.039). In conclusion, Lp N1115 was able to enhance the content of *Lactobacillus* and maintain fecal pH levels. Its beneficial effects on gut development were more obvious in 6–12-month-old infants.

## 1. Introduction

Cesarean section (C-section) is one of the most common obstetrical procedures, and China is among the countries with the highest C-section rates in the world [1]. Previous research showed that the composition of the intestinal microbiota in infants delivered vaginally is similar to that in the mother’s vagina, but the colonization and dominance of *Bifidobacteria* and *Lactobacillus* in the intestine of infants delivered by C-section occurs later [2]. This is closely related to metabolism, immunity, intestinal diseases, allergies, neuropsychiatric growth disorders, obesity, diabetes, and other chronic diseases in adulthood [3,4,5,6,7]. Thus, dysbacteriosis of the early intestinal flora that accompanies this procedure has gradually been recognized as an important public health problem [8].

Nevertheless, C-section is still a necessary and commonly used measure in obstetrics. In this context, preventing or reducing the destructive effects of the early establishment of bacteria is crucial for the healthy development of infants. The establishment of the intestinal microecology is a slow and complex dynamic process [9]. Given the dynamic nature of microbiota development with age, the first 1000 days of life—which includes gestation and the first two years of life—are seen as a window of opportunity to compensate for early microbial imbalance [10,11,12]. With the detection of *Lactobacillus*, Bifidobacterium and other microorganisms in human milk, early preventive probiotic supplementation for infants and young children has gradually become an international trend.

Tight junctions (TJs) [13] between intestinal epithelial cells in infants and young children and other immune factors are not yet mature. The amount of antibodies produced by the mucosa accounts for about 75% of total antibodies in the body, and the content of secreted immunoglobulin A (sIgA) is the highest [14]. sIgA can effectively neutralize bacterial toxins, pathogens, and enzymes to form antigen–antibody complexes. It can also stimulate the secretion of mucin and accelerate the flow of the mucus layer, thus accelerating the efflux of toxic substances such as bacteria and endotoxins and preventing the adhesion of viruses, bacteria, and antigens [15].

Fecal calprotectin (FC) and α1-antitrypsin (AAT) can be used to characterize intestinal maturity in infants. Calprotectin is commonly expressed in neutrophils and macrophages and is widely distributed in human cells, tissues, and body fluids [16]. The FC level in infants and young children is significantly higher than that in healthy adults, showing a downward trend with age, and is basically the same as that in adults by the age of 4 [17]. AAT is the most abundant serine protease inhibitor in the blood circulation system, can maintain the balance of proteases/anti-proteases, and has important anti-inflammatory and immune regulatory functions [18]. AAT is neither degraded by digestive enzymes nor reabsorbed by the intestine, so it theoretically parallels the intestinal loss of serum protein and can be used to evaluate gastrointestinal tract development and inflammation [19]. Viljanen and Mirva [20,21] carried out randomized controlled trials (RCTs) of *L. rhamnosus* (LGG) intervention in infants with atopic eczema/dermatitis syndrome or colic. The results showed that the level of ATT or FC in infants in the LGG intervention group was low, suggesting that probiotics could improve the intestinal permeability of infants to a certain extent.

*Lactobacillus* is one of the well-known and most studied probiotics and has a broad distribution in the human oral cavity, genitourinary tract, gastrointestinal tract, and milk [22]. Many studies have confirmed that *Lactobacillus* has multiple effects, such as regulating the intestinal microecology and promoting the development of the intestinal immune system. However, the mode of consumption, dosage, and delivery time of probiotics may have important impacts on their health effects. In addition, Szajewska [23] and other researchers reviewed 18 RCTs related to diarrhea in children. The results showed that LGG was more effective in European countries than in non-European countries, suggesting that the same probiotics might have different effects on people in different regions.

*Lactobacillus paracasei* N1115 (Lp N1115) was isolated from fermented milk products that have long been consumed by herders in Mongolia [24] and has potential probiotic properties such as the ability to promote intestinal development [25], improve intestinal symptoms [26], and regulate immunity [27]. However, the current research on Lp N1115 is mostly conducted on animals or in adults, and fewer trials are conducted on infants and young children. An RCT conducted in Ireland has confirmed that probiotic supplementation with Lp N1115 was well tolerated by the young children but has little effect on some other indicators of gut development [28]. We have confirmed that the administration of Lp N1115 is well tolerated in Chinese children [29], but the effectiveness of Lp N1115 on gut health among Chinese toddlers is still unclear. This study aimed to investigate the efficacy of Lp N1115 as a probiotic on immunomodulatory and gut microbial composition in Chinese infants and toddlers born by C-section.

## 2. Materials and Methods

The detailed study design and CONSORT flow diagram has been previously reported in detail [29] and will be described briefly here.

### 2.1. Study Design

**Figure 1:**
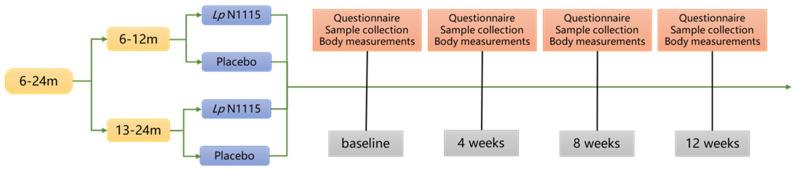
Experimental procedure.

This study was designed as a single-center, randomized, triple-blind placebo-controlled trial and was conducted at Xuchang Maternal and Child Health Hospital, Henan Province, China, from January 2020 to April 2021. Healthy infants born by C-section were recruited at the age of 6–24 months and divided into two age groups: 6–12 months and 13–24 months. After consent was obtained from their legal guardians, infants and toddlers were randomly assigned to either the experimental group (Lp N1115 group) or the placebo-control group, and a total of four main visits were conducted with an intervention duration of 12 weeks (Figure 1).

During the 12 weeks of the intervention, participants consumed a packet of Lp N1115 bacterial powder (2 × 10^10^ colony-forming units [CFU]/g) or a placebo (maltodextrin) in the same package every day. Compliance with the protocol was considered to be when ≥85% of the powder packages were brought back empty.

### 2.2. Participants

The criteria for subject inclusion were: (a) delivered at full-term (≥37 weeks); (b) aged 6–24 months; and (c) delivered by C-section. We excluded those infants who had taken probiotics or prebiotics within 2 weeks before the intervention or who had taken milk powder containing probiotics or used antibiotics within 3 months before the experiment. Additionally, those who were suffering from serious acute or chronic diseases (cardiovascular disease, gastrointestinal disease, endocrine disease, immune system disease, metabolic disease, etc.) and using drugs that may interfere with the experimental results were not included in the trial. Fulfillment of the inclusion and exclusion criteria was validated by the investigator and appointed research nurse.

### 2.3. Biological Sample Collection and Detection

#### 2.3.1. Saliva Sample Collection

The collection of saliva samples was completed by uniformly trained doctors or nurses in the hospital on the day the subjects were recruited or followed up. All saliva samples were collected between 9 a.m. and 11 a.m. A saliva sample was collected by inserting a cotton swab into the subject’s mouth and letting it rest on the floor of the mouth for 30 s. Immediately after collection, the swab filled with saliva was placed into the tube insert of a salivette collection device (Salivette Cortisol, code blue; 51.1534.500, Sarstedt, Sinnottstown Lane, Drinagh, Wexford, Ireland). Samples were centrifuged by the research nurse/assistant at 1000× *g* for 5–10 min at 4 °C. Saliva was recovered and pooled outside the tube insert within the salivette collection device. Once finished, the swab could be discarded. Saliva was aspirated into sterile Eppendorf tube(s) (Biosphere SafeSeal Tube 1.5 mL; 72.706.200, Sarstedt, Sinnottstown Lane, Drinagh, Wexford, Ireland) and stored in a −80 °C freezer.

#### 2.3.2. Fecal Sample Collection

Fecal samples were collected from infants at home by their parents or guardians during the 24 h before the baseline and at week 4, 8, and 12 visits. Samples were collected with a scoop attached to a sterile plastic collection tube. After collection, the tubes were immediately stored in a −20 °C freezer, transported to the laboratory within 1 day, and stored at −80 °C until processing.

### 2.4. Sample Measurement and Sequencing

All frozen samples were shipped on dry ice and entrusted to the Shanghai Institute of Quality Inspection and Technical Research (SQI) for unified testing.

An enzyme-linked immunosorbent assay (ELISA) kit (Cusabio; CSB-E09235h, 96T, Houston, TX, USA) was used to determine sIgA levels in saliva and fecal samples. Liquid chromatography-tandem mass spectrometry (LC-MS/MS) was used to determine cortisol levels in saliva samples. The pH of fecal samples was detected with a solid and semi-solid pH meter. An FC test kit (immunofluorescence chromatography, Guangzhou Fengrun Biotechnology Co., Ltd., Guangzhou, China) in combination with a dry fluorescent immunoanalyzer (model FR-101) was used to detect FC. The fecal AAT content was determined with an α1-antiprotease assay kit (immune transmission turbidimetry, Weifang 3D Biology, Weifang, China).

The 16S rDNA amplification method was used to amplify the v3–v4 variable region of 16S rDNA in samples using specific primers, construct a high-throughput sequencing library, and analyze the sequence of the variable region so as to identify the microbial composition and abundance. The sequencing process included sample DNA extraction and quality inspection, polymerase chain reaction (PCR) amplification, library preparation and detection, Miseq sequencing, and sequencing data quality control and optimization. The valid sequences obtained by sequencing on the Illumina MiSeq platform were clustered into operational taxonomic units (OTUs). OTUs were clustered at 97% sequence similarity, and the species distribution information was obtained based on Silva_138 database annotation. OTU tables were normalized to account for differences in sequence depth.

### 2.5. Statistical Analysis

The statistical analyses were performed using a per-protocol (PP) approach. Data were summarized as mean ± SD or median and interquartile range (IQR). Using the World Health Organization (WHO) Anthro software, the height-for-age Z-score (HAZ), weight-for-age Z-score (WAZ), and head circumference-for-age Z-score (HCZ) were calculated.

Student’s *t*-tests or Mann–Whitney U tests were used for continuous normal or non-normal variables, and chi-squared tests for categorical variables. To explore the impact of intervention measures on the subjects’ biochemical indicators, a stratified analysis and mixed linear effect model was used. Subjects were divided by age (6–12 and 13–24 months). Baseline values, group, intervention time, and the group × time interaction were used as fixed terms, and individuals were used as random terms to construct linear or generalized linear mixed-effects models to analyze the effects of the intervention on the outcomes.

Analysis of α and β diversity was used to explore the changes in species structure and abundance of intestinal flora before and after the intervention. Spearman’s rank correlation was used to analyze the association between *Lactobacillus* abundance and biochemical indicators.

Statistics analyses were performed using R statistical software (version 4.1.0) and the threshold for significance was set at *p* < 0.05.

## 3. Results

### 3.1. Study Population

A total of 247 infants aged 6–24 months and delivered by C-section were recruited into the study. After the initial screening, 128 infants who did not meet the inclusion criteria were excluded, and the parents or guardians of another 10 refused to participate. Finally, the parents or guardians of 109 infants signed the informed consent form and were officially included in the study. During the follow-up, five participants in the test group withdrew from the study due to the COVID-2019 epidemic, allergies, time conflicts, etc. Three participants in the control group withdrew due to the impact of the epidemic and diarrhea, with an overall dropout rate of 7.3%. Most of the subjects withdrew within 4 weeks, and data filling was limited, so the principle of following the research protocol was adopted for analysis. Table 1 showed the baseline characteristics of the 101 children who completed the intervention. No significant differences were observed between the two groups (*p* > 0.05).

### 3.2. Fecal pH

**Figure 2:**
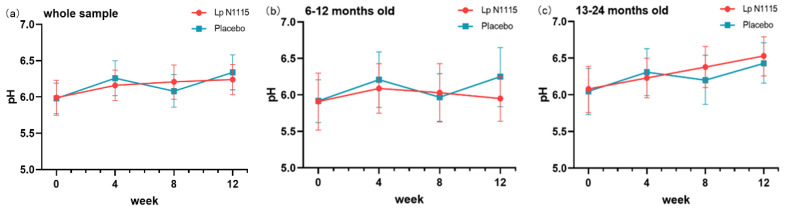
Fecal pH changes throughout the intervention. (**a**) Fecal pH changes of all participants; (**b**) Fecal pH changes of 6–12-month-old subgroup; (**c**) Fecal pH changes of 13–24-month-old subgroup.

After 12 weeks of intervention, the fecal pH of the two groups showed an upward trend with time (Figure 2a). Paired t-tests were used to analyze the changes in fecal pH from baseline at each time point, and Student t-tests were used to compare fecal pH between the two groups. The fecal pH in the control group increased from 5.98 at baseline to 6.34 (*p* = 0.003), while the fecal pH in the experimental group did not change significantly from baseline. In infants aged 6–12 months, the pH of the test group increased first and then decreased while that of the control group fluctuated, but the difference between the groups was not significant (Figure 2b). In infants aged 13–24 months, the pH of the test group and the control group increased from baseline after 12 weeks of intervention (Figure 2c, Lp N1115 group: *p* = 0.021; placebo group: *p* = 0.019). The mixed effect model did not detect any difference in the whole intervention stage.

### 3.3. Saliva Cortisol

**Figure 3:**
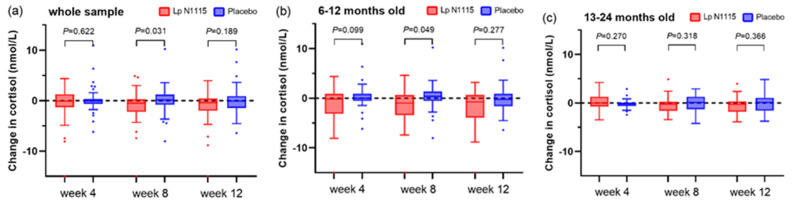
Changes in salivary cortisol from baseline. (**a**) salivary cortisol changes of all participants; (**b**) salivary cortisol changes of 6–12-month-old subgroup; (**c**) salivary cortisol changes of 13–24-month-old subgroup.

The Wilcoxon signed-rank test was used to analyze the changes in saliva cortisol from baseline at each time point within the group, and the Mann–Whitney U test was used to analyze the differences in the changes in saliva cortisol at each time point between the two groups. As shown in Figure 3a, at weeks 8 and 12 of the intervention, salivary cortisol decreased in the experimental group compared with baseline (week 8: Δ = −0.52 nmol/L, *p* = 0.022; week 12: Δ = −0.38 nmol/L, *p* = 0.023), while the control group showed little change compared with baseline, and the experimental group decreased more than the control group at week 8 (*p* = 0.031). The stratified analysis revealed similar results to those of the whole population for 6–12-month-old infants (Figure 3b), but there were no significant differences in 13–24-month-old infants (Figure 3c). The mixed-effects model adjusted for baseline, time, and group did not detect the presence of intervention effects, and the group × time interaction term was not significant.

### 3.4. sIgA

**Figure 4:**
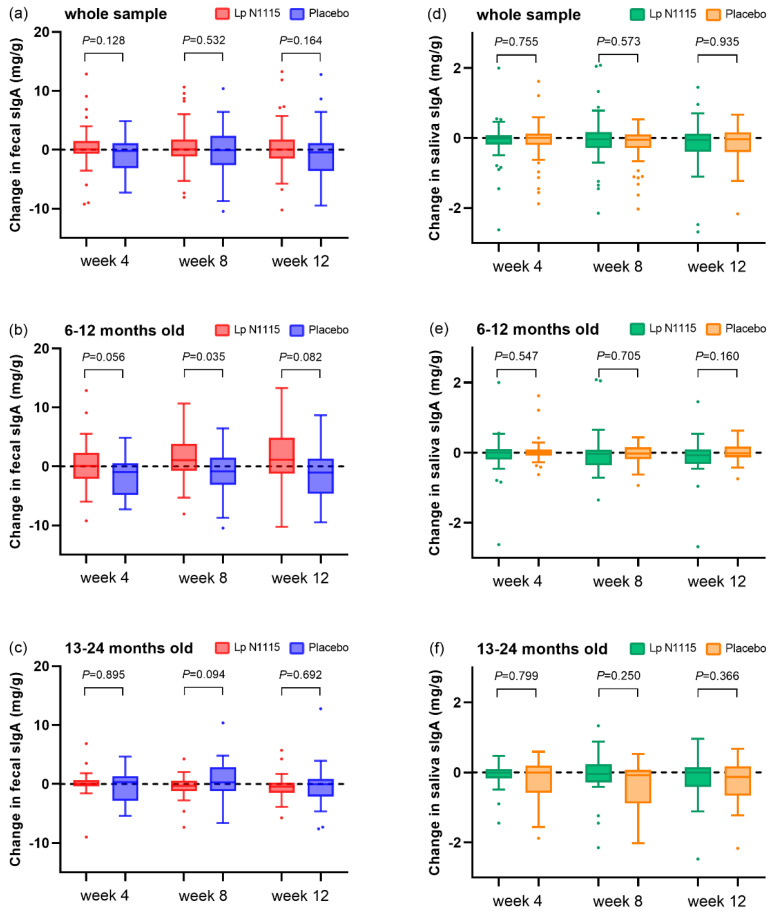
Changes in sIgA in feces and saliva compared with baseline. (**a**) fecal sIgA changes of all participants; (**b**) fecal sIgA changes of 6–12-month-old sub-group; (**c**) fecal sIgA changes of 13–24-month-old subgroup; (**d**) saliva sIgA changes of all participants; (**e**) saliva sIgA changes of 6–12-month-old sub-group; (**f**) saliva sIgA changes of 13–24-month-old subgroup.

The results of the sIgA analysis of infants’ feces and saliva are shown in Figure 4. During the intervention, the sIgA of infants’ stool in the Lp N1115 group increased while that in the placebo group decreased, but the difference was not significant (Figure 4a). When stratified by age, the results showed that fecal sIgA increased by 0.06, 1.08, and 1.12 mg/g in 6–12-month-old infants compared with baseline at the three follow-ups, respectively, while it decreased by 1.00, 0.88, and 1.02 mg/g, respectively, in the control group (Figure 4b). The mixed-effects model showed a significant intervention effect of Lp N1115 in 6–12-month-old infants (*p* = 0.044). There was no significant difference in 13–24-month-old infants (Figure 4c).

Analysis of salivary sIgA levels revealed that in the all participants and the 13–24-month-old subgroups only, sIgA in the control group was significantly decreased from baseline at the 8th week (Figure 4d–f, all participants: *p* = 0.045; 13–24-month-old subgroup: *p* = 0.028). The change in salivary sIgA in the experimental group relative to baseline was not significant.

### 3.5. FC and AAT

**Figure 5:**
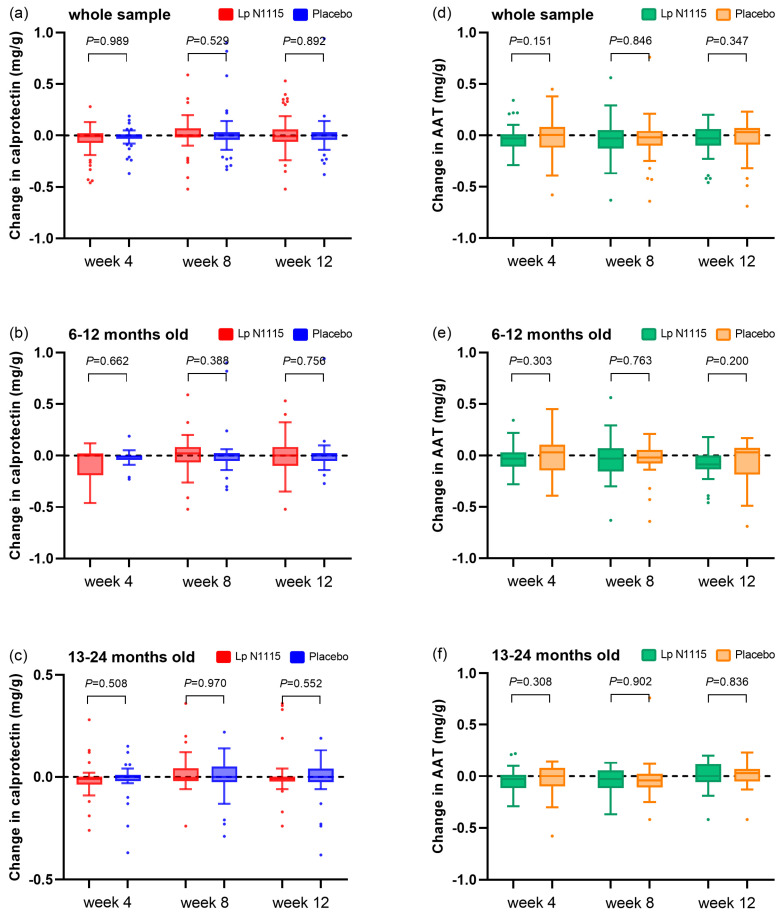
Changes in FC and AAT compared with baseline. (**a**) FC changes of all participants; (**b**) FC changes of 6–12-month-old sub-group; (**c**) FC changes of 13–24-month-old subgroup; (**d**) AAT changes of all participants; (**e**) AAT changes of 6–12-month-old sub-group; (**f**) AAT changes of 13–24-month-old subgroup.

There was no significant difference in the change in FC between the Lp N1115 and control groups before and after the intervention (Figure 5a–c). The changes in AAT are shown in Figure 5d–f. AAT decreased by 0.03 mg/g after 4 weeks of intervention in both the whole population and the 13–24-month-old subgroup in the Lp N1115 group (*p* = 0.018 and *p* = 0.046, respectively); at 12 weeks of intervention, the AAT decreased by 0.09 mg/g from baseline in the 6–12-month-old subgroup in the Lp N1115 group (*p* = 0.005). The change in the placebo group compared with the baseline was not significant. There were no significant differences according to any of the mixed-effects models.

### 3.6. Gut Microbiota

#### 3.6.1. Species Distribution

The representative sequences of OTUs were annotated and classified into 17 phyla, 22 classes, 63 orders, 119 families, and 308 genera. The abundance of *Firmicutes* in each group of samples was the highest, followed by *Bacteroidetes*, *Actinobacteria*, and *Proteobacteria*. These four phyla accounted for over 99% of all species, consistent with the currently known composition of human gut microbiota (Supplementary Figure S1).

Comparing the relative abundance and ratio (F/B) of *Firmicutes* and *Bacteroidetes* between two groups at different time points, it was found that the abundance of *Firmicutes* in the control group was significantly higher than that in the Lp N1115 group at week 8. There was no difference between the two groups at other time points, indicating that there was no significant change in the gut microbiota after intervention at the phylum classification level (Table 2). The distribution of the main species of the research subjects at the genus classification level is also similar (Supplementary Figure S2).

#### 3.6.2. α and β Diversity

We assessed changes in fecal bacterial community structure in response to the intervention. We noted an increase in α-diversity in the Lp N1115 group at the end of the intervention, but the control group only showed a significant change in the Shannon index before and after the intervention (Supplementary Table S1). The result of the stratified analysis is shown in Supplementary Figure S3. For the 6–12-month-old infants, the mixed-effects models showed significant group × time effects for the Shannon index (*p* = 0.023) and Simpson index (*p* = 0.012). The results for the 13–24-month-old subjects were similar to the whole population. However, we did not find any evidence demonstrating that β-diversity differed between the two groups at any time point using the Principal co-ordinates analysis (PCoA) and Partial least squares discriminant analysis (PLS-DA) (Supplementary Figure S4).

#### 3.6.3. Fecal *Lactobacillus*

**Figure 6:**
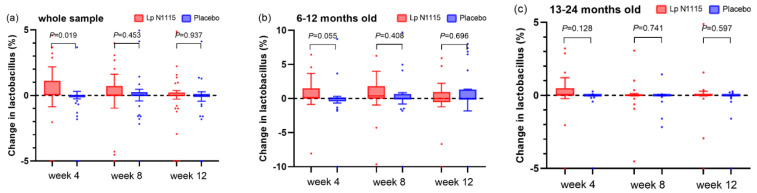
Changes in the relative abundance of *Lactobacillus* compared with baseline. (**a**) relative abundance of *Lactobacillus* changes of all participants; (**b**) relative abundance of *Lactobacillus* changes of 6–12-month-old subgroup; (**c**) relative abundance of *Lactobacillus* changes of 13–24-month-old subgroup.

Based on the results of the whole population and age-stratified analyses, the relative abundance of *Lactobacillus* in the Lp N1115 group showed a trend of increasing and then regressing to the baseline value at each time point, while the control group showed little change (Figure 6). At week 4, the increase in *Lactobacillus* relative to baseline was significantly higher in the experimental group than in the placebo group. The mixed-effect model did not detect the difference between groups during the intervention. Further analysis of the detection rate of fecal *Lactobacillus* showed a trend toward a higher detection rate of *Lactobacillus* in the Lp N1115 group than in the placebo group at weeks 8 (*p* = 0.089) and 12 (*p* = 0.059). The results of the mixed-effect model showed that the *Lactobacillus* species in the experimental group trended higher than that in the control group during the whole intervention stage (*p* = 0.050). After further adjustment for gender and baseline age, the results of the mixed-effects model showed a significant intervention effect (*p* = 0.039). No difference was found by stratified analysis (Supplementary Table S2).

### 3.7. Correlation between Fecal Lactobacillus and Biochemical Indexes

The correlation between the abundance of lactobacilli and each biochemical index was also stratified by age and time point of the intervention (Supplementary Table S3). Among 6–12-month-old infants, fecal and saliva sIgA correlated positively and significantly with the relative abundance of *Lactobacillus* after 12 weeks of intervention (r = 0.304 and r = 0.310, respectively). Additionally, from week 0 to week 8, the pH of infant stool correlated negatively with the abundance of *Lactobacillus* (r = −0.327). In 13–24-month-old subjects, there was a significant positive correlation between infant stool sIgA and *Lactobacillus* only at baseline.

## 4. Discussion

This study confirmed that Lp N1115 can help maintain the intestinal pH of infants aged 6–24 months after C-section, improve immune function, and promote the proliferation of *Lactobacillus*.

We found that the stool pH of the control group increased significantly after the intervention. Although the fecal pH of the test group increased, the change was not significant compared with baseline. Some researchers have reported that after an intervention with *B. lactis* Bb12 in premature infants, the fecal pH value was significantly lower than that in the placebo group [30]. However, no significant change in fecal pH value was detected in an intervention with healthy term infants with the same probiotics [31], suggesting that the intervention effect of probiotics varies greatly among different populations. It should be noted that the fecal pH test may be affected by various factors, such as the type of food consumed by the body, health status, and freezing and thawing of feces, and the results are relatively weak.

The intestinal microflora can affect human emotions and stress levels through the microbiota–gut–brain axis [32]. Cortisol is one of the most important glucocorticoids in the human body. The concentration of salivary cortisol is not affected by the flow rate of saliva and can fluctuate with different psychological states and emotions, which can effectively reflect the stress level of the body [33]. In a published cluster RCT, researchers used yogurt containing mixed probiotics (*Lactobacillus rhamnosus* yoba 2012 and *Streptococcus thermophilus* C106) or single probiotics (*Streptococcus thermophilus* C106) as an intervention with 262 children aged 4–7 years in two economically depressed schools in urban areas of Côte d’Ivoire. After one semester of intervention, it was found that the salivary cortisol of children who drank yogurt with mixed probiotics was significantly lower [34], suggesting that mixed probiotics reduced stress levels in children of lower socio-economic status. The results of this study showed that after the intervention, the salivary cortisol of infants in the Lp N1115 group was significantly lower than that at baseline, that is, the stress level was reduced, while the change in the control group was not significant. This decreasing trend was more obvious in infants aged 6–12 months. Although a single cortisol measurement cannot represent the whole day’s cortisol secretion, this study fixed the time window of saliva collection at each follow-up and intervened in multiple collections at different time points, which can be used for longitudinal analysis, and the results still have a certain value.

We found that the stool sIgA of the Lp N1115 intervention group showed an upward trend while that of the control group showed a downward trend, but only statistically significant differences were detected in the 6–12-month-old group, which may be due to factors such as weaning at 6–12 months and supplementary food addition, which accelerated the development of infant humoral immunity and which made them more susceptible to probiotics. The overall intervention effect on saliva sIgA was not obvious. However, the study of Brandtzaeg et al. suggests that the correlation between salivary sIgA and the intestinal sIgA reaction is weak [35]. Physiological conditions such as the salivary flow rate may affect the concentration of sIgA, and the characterization ability of the mucosal immune network of the body may be weaker than that of intestinal sIgA [36]. A study published in 2019 showed that after 132 infants aged 3.5–6 months were randomly treated with formula milk powder containing mixed probiotics or placebo for 4 weeks, the fecal sIgA level in the probiotic group was higher, and the saliva sIgA also showed an increasing trend [37], suggesting that the intervention effect of mixed probiotics is also more obvious for fecal sIgA.

Compared with infants delivered naturally, the delayed colonization of bifidobacterial and lactobacilli in infants delivered by C-section may affect intestinal development to a certain extent [38]. In this study, calprotectin and AAT in infant feces were detected to characterize infant intestinal maturity. Although the results showed that Lp N1115 had no significant effect on calprotectin or AAT in infant feces, previous researchers used LGG for an intervention with infants with atopic eczema/dermatitis syndrome or colic, and the results showed that the level of AAT or calprotectin in infants in the intervention group was low [20,21]. Mohan et al. [30] carried out a randomized double-blind controlled trial of *Bifidobacterium lactis* Bb12 in premature infants (90% of whom were delivered by C-section). The results showed that after 3 weeks of intervention, the calprotectin in the probiotic group was lower, and the difference was statistically significant, suggesting that the influence of probiotics on gastrointestinal development-related indicators may be more sensitive in newborns, premature infants, or other conditions causing a high level of inflammation in the body.

The infant intestinal flora is affected by many factors. A review published in 2021 further clarified that C-section has a certain negative impact on infant intestinal microecology balance and health outcomes [39]. Davis and other researchers summarized the effects of probiotic intervention on the microbial composition of healthy infants and children from birth to adolescence, and the results suggested that infants delivered by cesarean may benefit more than those delivered vaginally [40]. Intervention studies conducted by the Finnish scholar Korpela and others using mixed probiotics also confirmed that probiotic supplementation in early life can help infants and young children recover a normal flora and restore intestinal homeostasis after a C-section [41]. In a previous study in Ireland, researchers used Lp N1115 to intervene in infants after C-section for 8 weeks, and the research results confirmed that the bacteria can have a certain impact on the infant intestinal flora [28]. On the basis of this study, we used the same probiotics for an intervention with infants and children and extended the intervention time to 12 weeks. We collected fecal samples at multiple time points, at the baseline and the 4th, 8th, and 12th weeks. The results further verified that probiotics can regulate the intestinal microecology of infants and children to a certain extent. The detection rate of lactobacilli in infants in the test group increased significantly, and the abundance of lactobacilli showed a trend of first increasing and then decreasing within a low range, while the control group showed little change. The reason why the increase of Lactobacillus is not very great may be that the subjects are all over 6 months old. By this time, the intestinal flora of infants has developed, and there are relatively many influencing factors, such as diet, etc. Some scholars believe that the younger the age at which the probiotic intervention starts, the better the intervention effect will be [42].

In order to understand the relevant mechanisms linking changes in the bacterial flora composition and the health effects of probiotic intervention, this study attempts to analyze the relationship between the intestinal flora and biochemical indicators and diseases. The results showed that the correlation between *Lactobacillus* and sIgA was more obvious in infants aged 6–12 months. The above results showing such a correlation suggests that Lp N1115 intervention may increase the relative abundance of *Lactobacillus* and then improve the sIgA of infant stool, maintain fecal pH, etc. In addition, correlations among salivary sIgA, cortisol, AAT, and *Lactobacillus* are relatively weak, which may explain why these indicators changed little after Lp N1115 intervention.

The use of *Lactobacillus* in healthy cohorts is common, but available data are limited in terms of repeated measurements over the time and duration of probiotic administration. The Lp N1115 used in this study is a strain of local origin in China, and although it has been marketed for nearly a decade, the application of this strain to infants and children is still at an early stage. Overall, Lp N1115 has certain scientific research value and application prospects and is worthy of further exploration. Based on previous trials in Ireland [28], this study narrowed the age range of subjects while extending the duration of intervention and conducting monthly follow-up, which optimizes the research design and makes the research results more reliable. Preliminary research results have confirmed that the use of Lp N1115 could reduce the incidence rate of constipation and abdominal pain [29]. This study continued to explore the effect of Lp N1115 on the intestinal development of healthy Chinese infants. Saliva and fecal samples at different time points within the dry anticipation period were obtained and tested, providing a scientific basis for the rational use of Lp N1115 in infant food in the future.

This study has certain limitations. First, considering the particularity of infants and young children, this study has included infants and young children from the age of 6 months. Most of the study subjects had complementary foods added to their diets, and there were many confounding factors that may have covered up the intervention effect to a certain extent. In addition, to avoid invasive tests such as blood collection, only saliva and stool samples were collected in this study, and the biochemical indicators measured were of limited representativeness. This study is a preliminary exploration of the application of Lp N1115 in infants and young children, and a control group of infants delivered naturally and young children was not included in the design. The extrapolation of the results is limited, and the long-term impact of the Lp N1115 intervention cannot be obtained.

## 5. Conclusions

In conclusion, Lp N1115 was able to enhance the content of *Lactobacillus* and maintain fecal pH levels. Lp N1115 could increase fecal sIgA levels and, to some extent, reduce cortisol levels in infants and children, and its beneficial effects were more obvious in 6–12-month-old infants.

## Figures and Tables

**Table 1:** Study population characteristics.

	Lp N1115 (*n* = 51)	Placebo (*n* = 50)
**Gender**, *n* (%)		
Male	24 (47.1)	24 (48.0)
Female	27 (52.9)	26 (52.0)
**Age at enrolment**, *n* (%)		
6–12 months	25 (49.0)	25 (50.0)
13–24 months	26 (51.0)	25 (50.0)
**Solid food intake**, *n* (%)		
Yes	48 (94.1)	45 (90.0)
No	3 (5.9)	5 (10.0)
**Feeding type within 6 months after birth**, *n* (%)		
Exclusive breastfeeding	29 (56.9)	26 (52.0)
Mixed feeding	21 (41.2)	23 (46.0)
Formula feeding	1 (2.0)	1 (2.0)
**Body measurements**, mean ± SD		
HAZ	0.30 ± 0.85	0.32 ± 1.02
WAZ	0.32 ± 1.46	−0.01 ± 1.24
HCZ	−0.37 ± 1.34	−0.44 ± 1.27
**Fecal pH**, mean ± SD	6.16 ± 0.75	6.26 ± 0.84
**Biochemical indicators at baseline**, median (25th, 75th)		
Saliva cortisol, nmol/L	2.12 (1.14, 3.44)	1.54 (1.02, 2.64)
Saliva sIgA, mg/g	0.39 (0.24, 0.74)	0.35 (0.13, 0.78)
Fecal sIgA, mg/g	2.93 (1.42, 5.65)	3.36 (1.33, 7.31)
Calprotectin, mg/g	0.03 (0.02, 0.10)	0.02 (0.01, 0.09)
α1-antitrypsin, mg/g	0.34 (0.27, 0.46)	0.33 (0.25, 0.40)

WAZ: weight-for-age Z-score, HAZ: height-for-age Z-score, HCZ: head circumference-for-age Z-score.

**Table 2:** The relative abundance and ratio (F/B) of Firmicutes and Bacteroidetes.

Species	Weeks	Lp N1115 Group, Median (25th, 75th)	Control Group, Median (25th, 75th)	*p* ^a^
**Firmicutes**	0	42.4 (30.0, 57.6)	49.9 (35.4, 66.1)	0.120
	4	47.5 (30.8, 60.1)	55.3 (34.6, 68.6)	0.092
	8	41.6 (30.7, 55.1)	51.2 (40.4, 68.1)	0.010
	12	44.0 (31.9, 58.7)	49.5 (39.3, 62.8)	0.150
**Bacteroidetes**	0	21.4 (0.03, 35.1)	13.3 (0.1, 31.6)	0.984
	4	16.5 (0.03, 37.6)	12.0 (30.0, 57.6)	0.994
	8	20.0 (0.05, 45.3)	23.5 (0.9, 36.0)	0.676
	12	23.1 (0.13, 40.1)	26.8 (3.5, 40.7)	0.534
**F/B**	0	2.4 (1.2, 528.2)	5.7 (1.5, 396.6)	0.753
	4	3.1 (1.1, 363.0)	5.2 (1.5, 197.9)	0.726
	8	2.7 (0.8, 554.4)	2.2 (1.2, 74.6)	0.699
	12	2.0 (1.1, 147.7)	2.0 (0.9, 18.2)	0.632

^a^ Mann–Whitney U test.

## Data Availability

The data presented in this study are available on request from the corresponding author. The data are not publicly available due to privacy and ethical restrictions.

## Supplementary Materials

The following supporting information can be downloaded at: https://www.mdpi.com/article/10.3390/nu15081970/s1, Table S1: The changes of α diversity indexes by all participants, Table S2: Detection rate of fecal Lactobacillus, Table S3: Correlation between fecal Lactobacillus and biochemical indexes. Figure S1: The changes in relative abundance of species at the phylum level; Figure S2: The changes in relative abundance of species at the genera level; Figure S3: The changes of α diversity indexes by age; Figure S4: PCoA and PLS-DA models for differences in fecal microbiota in all participants.
