# Supplementary material for: Effect of Lacticaseibacillus paracasei N1115 on Immunomodulatory and Gut Microbial Composition in Young Children: A Randomized, Placebo-Controlled Study
Source: Nutrients. 2023 Apr 19;15(8):1970. doi: 10.3390/nu15081970 (PMC10145370; doi:10.3390/nu15081970)
Supplement: Supplementary file 1 [file nutrients-15-01970-s001.zip › nutrients-2316512-supplementary.pdf]

Supplementary Table S1. The changes of α diversity indexes by all participants

α diversity	Weeks	Lp N1115 group		Control group		P [b]	P [c]		
		median (25th,75th)	P [a]	median (25th,75th)	P [a]		group	time	group×time
Chao 1	0	326.7 (215.8, 421.3)	-	275.3 (229.7, 485.1)	-	0.860	0.324	0.526	0.572
	4	333.4 (226.9, 511.9)	0.087	309.1 (226.1, 478.4)	0.257	0.730			
	8	336.5 (198.1, 510.3)	0.086	317.0 (236.7, 436.8)	0.269	0.903			
	12	367.4 (215.4, 522.0)	**0.005**	364.1 (236.4, 438.5)	0.131	0.555			
ACE	0	324.3 (202.4, 408.4)	-	277.2 (228.8, 448.0)	-	0.930	0.358	0.405	0.600
	4	337.4 (218.8, 507.1)	0.126	304.3 (223.9, 492.1)	0.290	0.767			
	8	324.3 (202.1, 503.2)	0.157	321.0 (243.2, 431.7)	0.318	0.962			
	12	365.7 (219.2, 512.5)	**0.002**	364.0 (245.3, 455.0)	0.149	0.592			
Shannon	0	3.0 (2.2, 3.4)	-	3.0 (2.4, 3.6)	-	0.447	0.785	0.122	0.352
	4	3.1 (2.4, 3.7)	0.062	3.1 (2.6, 3.7)	0.553	0.634			
	8	3.2 (2.5, 3.6)	**0.028**	3.1 (2.5, 3.8)	0.342	0.744			
	12	3.2 (2.5, 3.8)	**0.016**	3.3 (2.7, 3.8)	**0.026**	0.472			
Simpson	0	0.12 (0.06, 0.28)	-	0.10 (0.06, 0.16)	-	0.299	0.658	0.602	0.083
	4	0.10 (0.05, 0.17)	0.052	0.09 (0.05, 0.17)	0.858	0.436			
	8	0.09 (0.06, 0.19)	**0.026**	0.09 (0.06, 0.17)	0.668	0.596			
	12	0.10 (0.05, 0.21)	**0.023**	0.09 (0.05, 0.14)	0.100	0.403			

[a] Wilcoxon signed rank test, [b] Mann Whitney U test, [c] mixed linear effect model

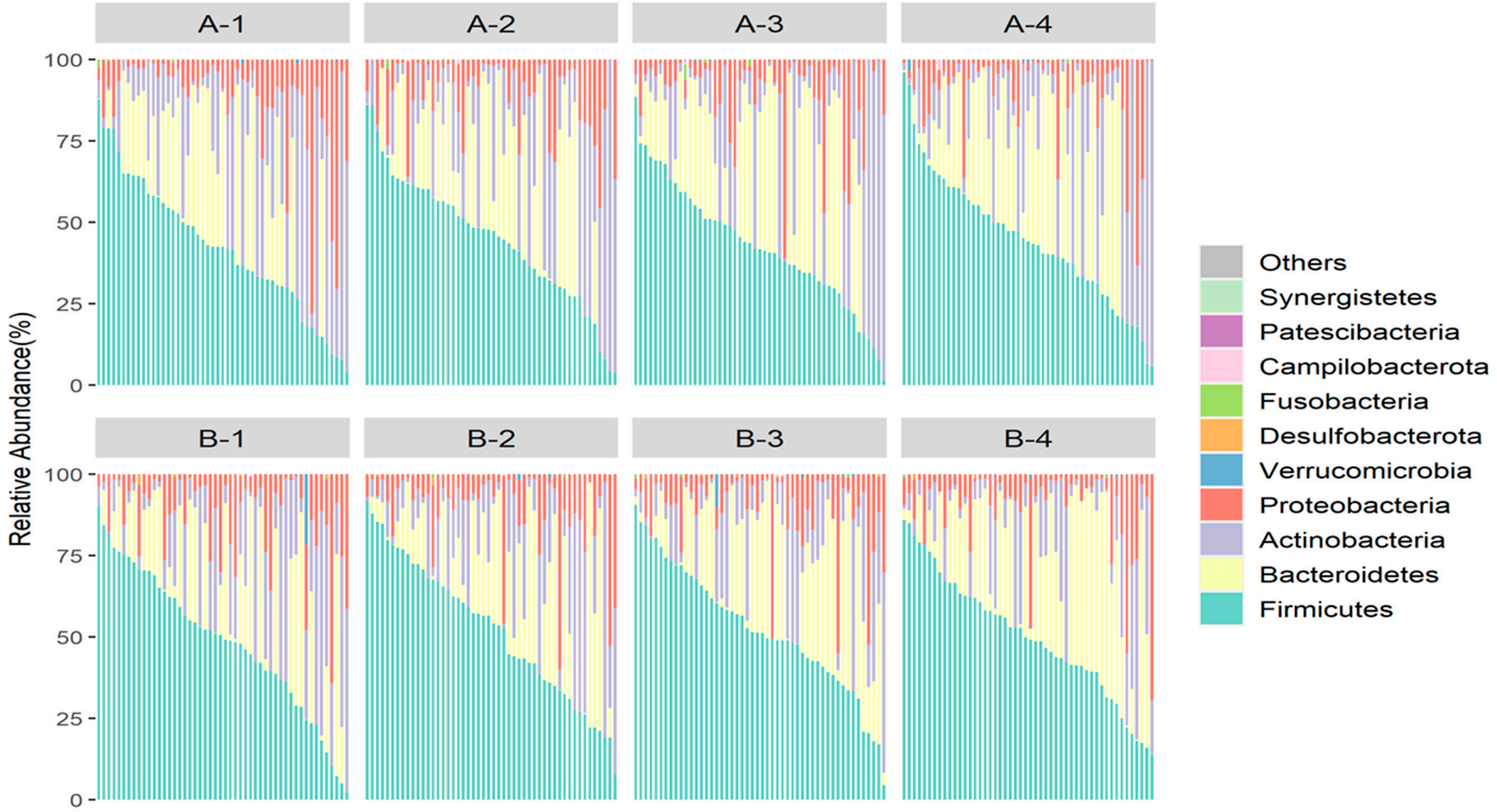

Supplementary Figure S1. The changes of relative abundance of species at phylum level

A-1: Lp N1115 group, 0weeks, A-2: Lp N1115 group, 4weeks, A-3: Lp N1115 group, 8weeks, A-4: Lp N1115 group, 12weeks, B-1: control group, 0weeks, B-2: control group, 4weeks, B-3: control group, 8weeks, B-4: control group, 12weeks

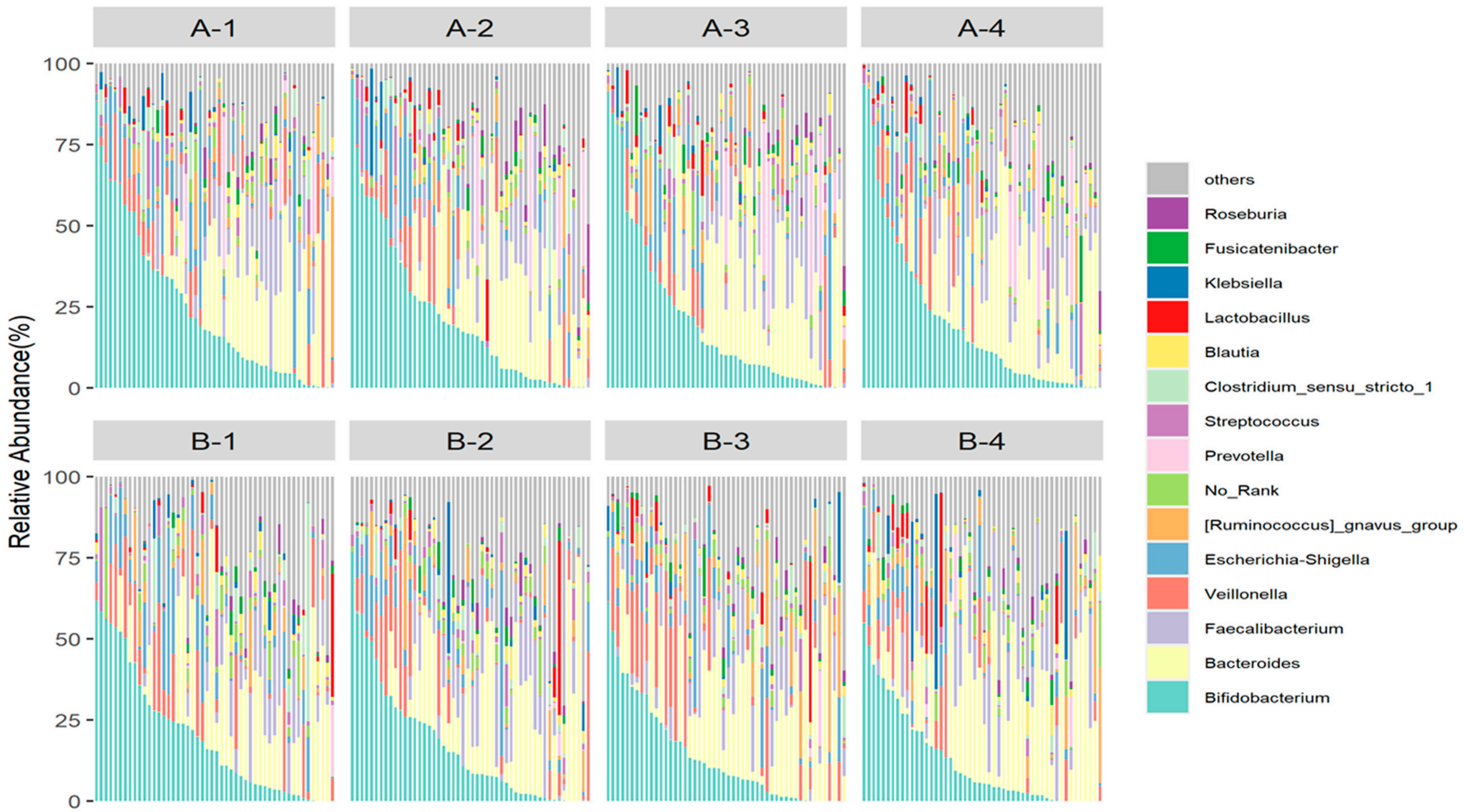

Supplementary Figure S2. The changes of relative abundance of species at genera level

A-1: Lp N1115 group, 0weeks, A-2: Lp N1115 group, 4weeks, A-3: Lp N1115 group, 8weeks, A-4: Lp N1115 group, 12weeks, B-1: control group, 0weeks, B-2: control group, 4weeks, B-3: control group, 8weeks, B-4: control group, 12weeks

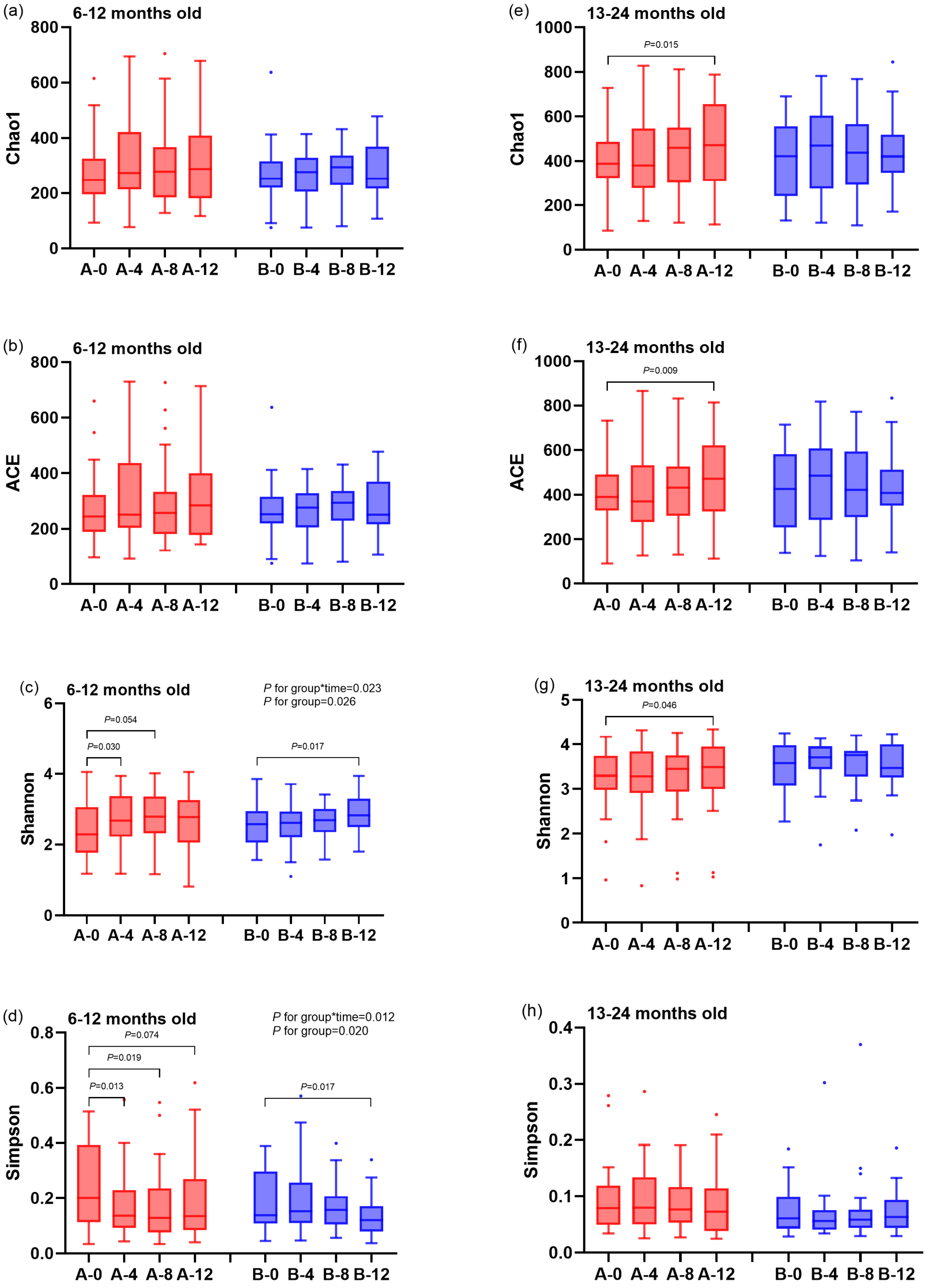

Supplementary Figure S3. The changes of α diversity indexes by age

A-0: Lp N1115 group, 0weeks, A-4: Lp N1115 group, 4weeks, A-8: Lp N1115 group, 8weeks, A-12: Lp N1115 group, 12weeks, B-0: control group, 0weeks, B-4: control group, 4weeks, B-8: control group, 8weeks, B-12: control group, 12weeks; P: Mann Whitney U test; P for group, P for group*time: mixed effect model

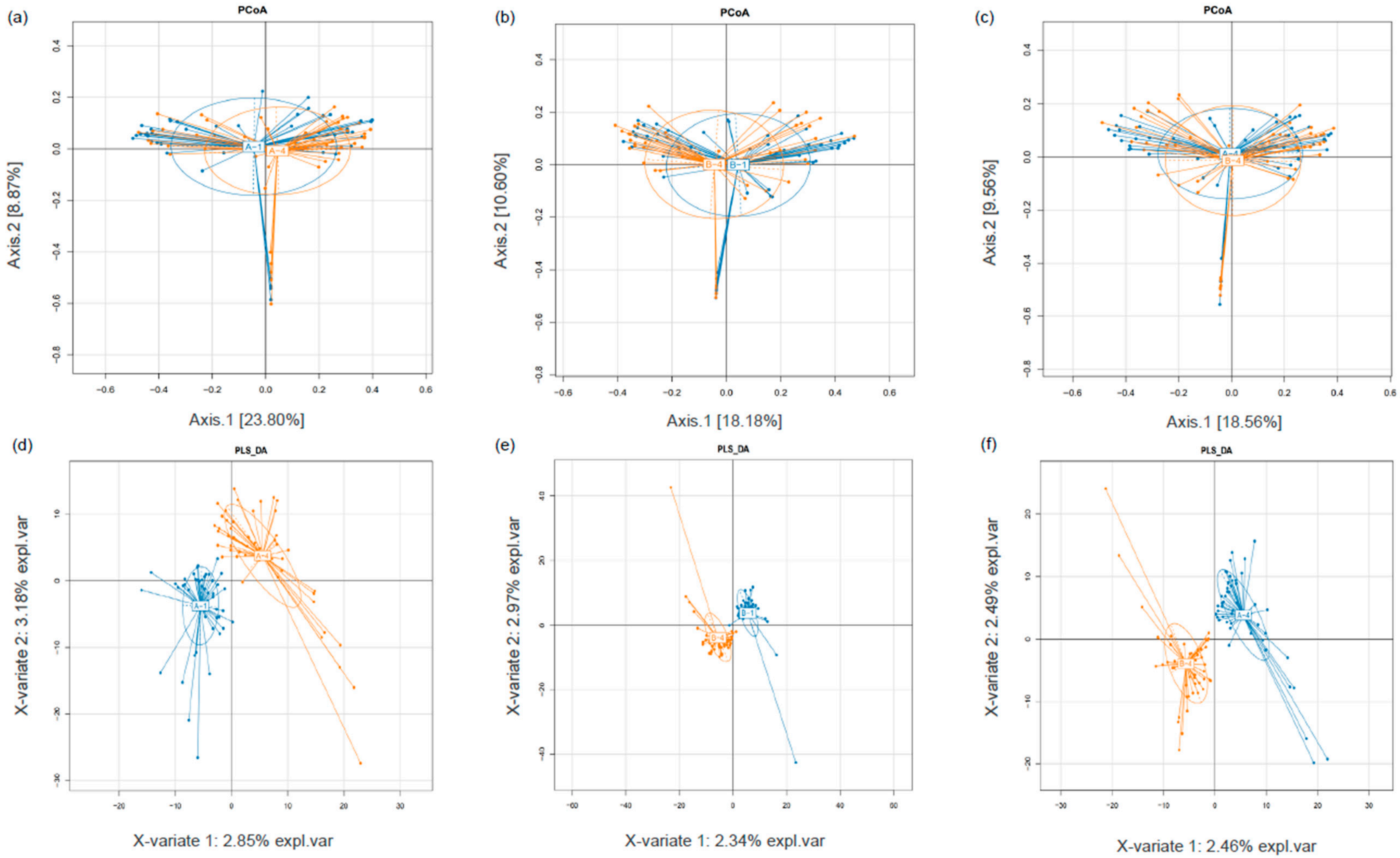

Supplementary Figure S4. PCoA and PLS-DA models for differences in fecal microbiota in all participants

A-1：Lp N1115 group, baseline (0 weeks); A-4: Lp N1115 group,12 weeks; B-1: control group,12 weeks; B-4: control group,12 weeks

Expl.var：explanation of variance

Supplementary Table S2. Detection rate of fecal Lactobacillus

Weeks	Lp N1115	Placebo	P [a]	P [b]		
				group	time	group*time
**All participants**						
0	28 (54.9)	22 (44.0)	0.273	**0.050**	**0.007**	0.740
4	37 (72.5)	30 (60.0)	0.182			
8	34 (66.7)	25 (50.0)	0.089			
12	31 (60.8)	21 (42.0)	0.059			
**6–12 months old**						
0	16 (54.0)	15 (60.0)	0.771	0.124	**0.024**	0.915
4	20 (80.0)	17 (68.0)	0.333			
8	19 (76.0)	15 (60.0)	0.225			
12	17 (68.0)	12 (48.0)	0.152			
**13–24 months old**						
0	12 (46.2)	7 (28.0)	0.180	0.155	0.107	0.821
4	17 (65.4)	13 (52.0)	0.332			
8	15 (57.7)	10 (40.0)	0.206			
12	14 (53.8)	9 (36.0)	0.200			

[a]chi-square test, [b]mixed effect model

Supplementary Table S3. Correlation between fecal Lactobacillus and biochemical indexes

weeks	6–12 months old				13–24 months old			
	0	4	8	12	0	4	8	12
**pH**	-0.330*	-0.327*	-0.478*	-0.258	-0.340*	-0.297*	-0.130	0.020
**Saliva Cortisol**	-0.147	-0.045	-0.021	-0.156	0.125	0.138	0.298*	0.152
**Saliva sIgA**	0.225	-0.001	0.009	0.310*	0.042	0.033	-0.095	-0.135
**Fecal sIgA**	0.059	0.226	0.219	0.304*	0.319*	0.163	0.043	-0.190
**FC**	0.148	0.124	0.259	0.222	0.227	-0.120	0.215	-0.228
**AAT**	0.130	-0.035	-0.190	0.024	-0.270	0.093	-0.019	-0.326*

*$p < 0.05$
